# Divergent manifestations and management of GISTs of the small intestine: a case series

**DOI:** 10.1093/jscr/rjag134

**Published:** 2026-03-07

**Authors:** Devendra Bist, Roshan Ghimire

**Affiliations:** Department of GI & General Surgery, Kathmandu Medical College and Teaching Hospital, Sinamangal, PO Box 12127, Kathmandu, Nepal; Department of GI & General Surgery, Kathmandu Medical College and Teaching Hospital, Sinamangal, PO Box 12127, Kathmandu, Nepal

**Keywords:** gastrointestinal stromal tumor, GIST, small intestine, ileal GIST, jejunal GIST, duodenal GIST, segmental bowel resection, imatinib, surgery

## Abstract

Gastrointestinal stromal tumours (GISTs) of the small intestine are rare but clinically significant mesenchymal neoplasms, often presenting with gastrointestinal bleeding, obstruction, or perforation. This retrospective case series analyses six cases managed over 18 months, highlighting their diverse presentations, diagnostic challenges, and surgical outcomes. Gastrointestinal bleeding was the most common symptom (*n* = 4), followed by obstruction (*n* = 2) and perforation (*n* = 1), with one case detected incidentally. Contrast-enhanced computed tomography was crucial in preoperative diagnosis, revealing exophytic, or intraluminal masses. All patients underwent surgical resection, including segmental bowel resection with anastomosis or wedge resection, with histopathology confirming GIST in all cases. Postoperative recovery was uneventful in most patients, except for one case of transient paralytic ileus requiring intensive care. This study highlights the diagnostic challenges of small intestinal GISTs and emphasizes the role of early imaging and timely surgical intervention in improving outcomes. Further research is needed to refine treatment strategies and long-term prognostication.

## Introduction

Gastrointestinal stromal tumours (GISTs) are the most common mesenchymal tumours in the digestive tract, making up ~80% of these cases. While they are relatively rare, they account for 0.1% to 3% of all gastrointestinal cancers [1]. They are diagnosed in ~10–15 people per million worldwide each year and most often affect individuals in their 60s and 70s, with men and women being equally at risk [2–4]. GISTs were first identified as a distinct type of tumour in 1983 when researchers discovered that they contained characteristics of both smooth muscle and neural cells [5]. Later, Hirota *et al*. found that most GISTs have mutations in the KIT proto-oncogene (cluster of differentiation 117), causing continuous activation of the KIT receptor tyrosine kinase, which drives tumor growth [6]. GISTs can develop anywhere along the gastrointestinal tract, with the stomach being the most common site (60%), followed by the small intestine (20%–30%) [3, 7, 8]. Diagnosing small bowel GIST is challenging as these tumours are rare, and their symptoms are often subtle or nonspecific, making them easy to overlook, also the small intestine area is difficult to assess via conventional endoscopies like upper and lower GI endoscopies [9, 10].

## Case descriptions

**Figure 1:**
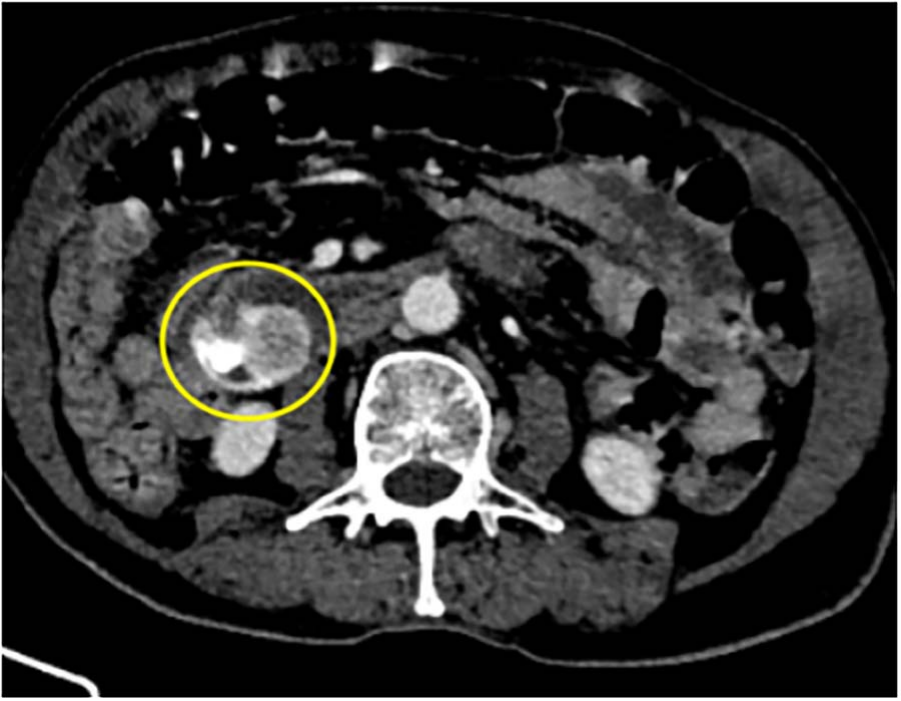
88Y/M with 2 episodes of black tarry stool, axial view of the contrast-enhanced computed tomography (CECT) abdomen showing a heterogenous mass with extravasation of contrast material, signifying the source of hemorrhage.

**Figure 2:**
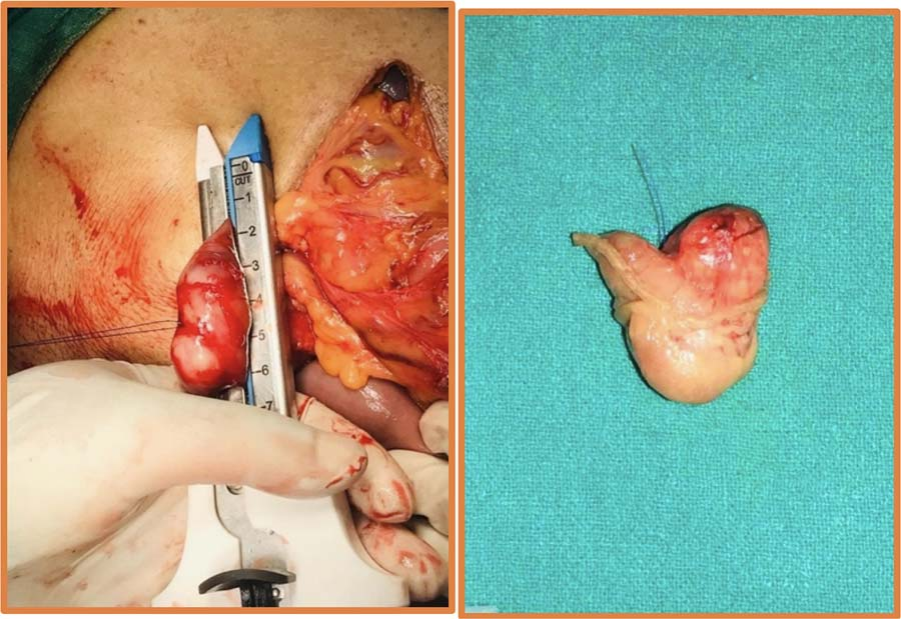
Intraoperative (left) and postoperative resected specimen (right) of 72Y/M with asymptomatic, incidental duodenal GIST.

**Figure 3:**
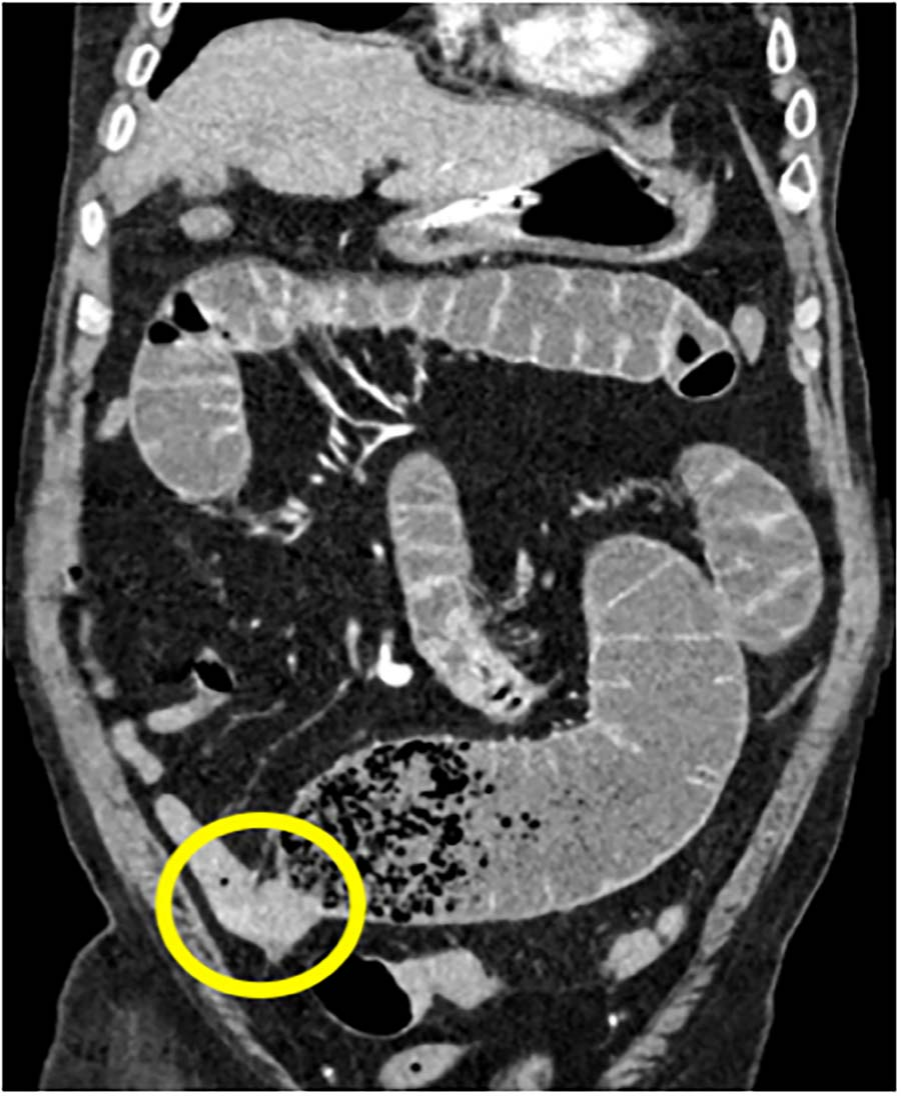
75Y/M with upper abdominal pain, vomiting, abdominal distention, coronal view of the CECT abdomen showing intraluminal mass in the distal jejunum, causing dilatation of the bowel proximall.

**Figure 4:**
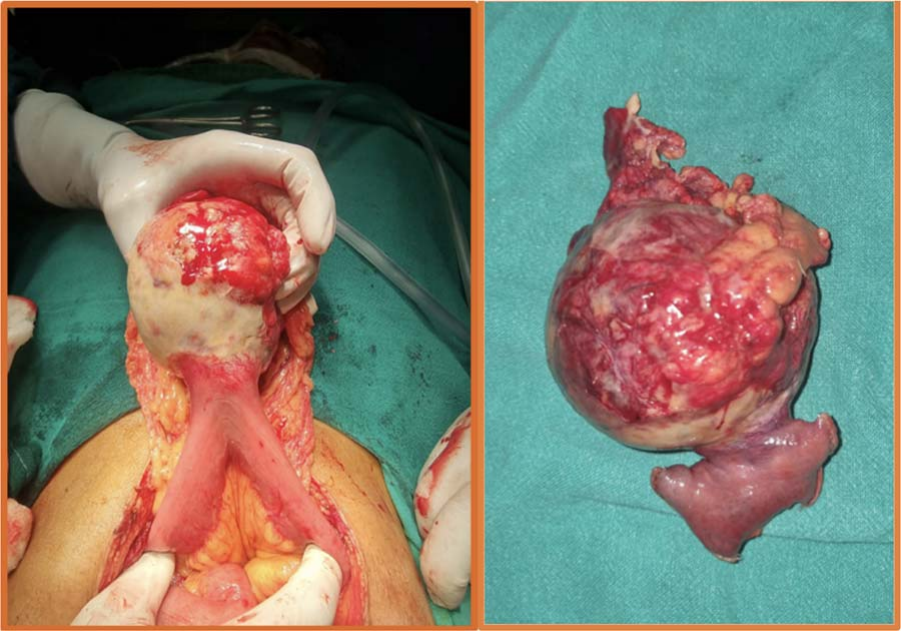
Intraoperative (left) and postoperative resected specimen (right) of a 70/female with bleeding jejunal GIST underwent open segmental resection of jejunal GIST with end to end jejunojejunal anastomosis.

**Figure 5:**
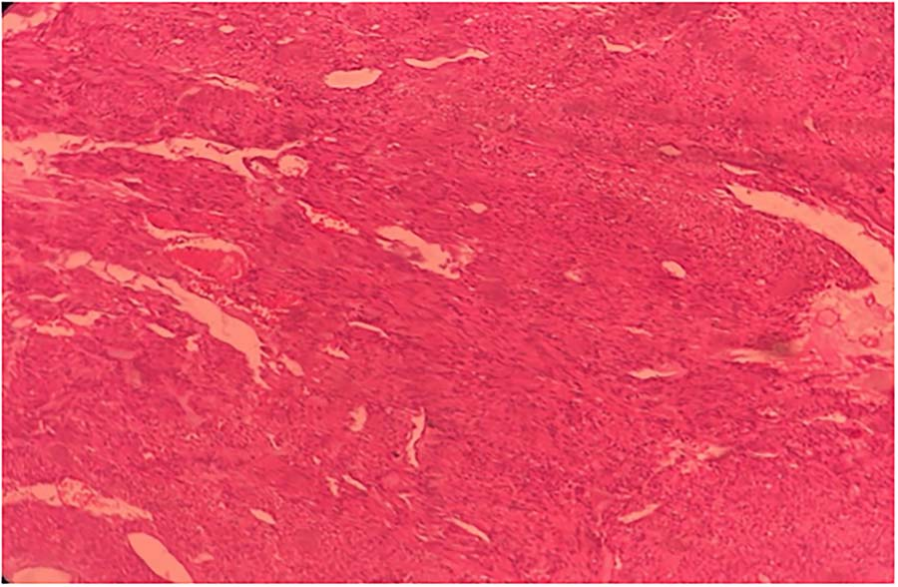
Histopathological slide of the resected specimen of 55Y/M with jejunal GIST.

Our case series includes six patients from ages 55–88 years diagnosed with small intestinal GISTs, presenting with varied clinical manifestations between 1 January 2023 and 31 June 2024. The most common presentation was gastrointestinal bleeding in three cases (Figs 1 and 4), followed by one case each of intestinal obstruction (Fig. 3) and perforation (Fig. 5), both of which presented with abdominal pain. One patient was incidentally diagnosed (Fig. 2) during imaging for unrelated symptoms. Tumour locations included the jejunum in three cases, duodenum in two cases, and one in ileum. Tumour sizes ranged from 2 to 15 cm. Surgical interventions included segmental bowel resections with anastomosis for jejunal and ileal tumours, and wedge resections for duodenal lesions. Postoperative recovery was generally uneventful, except for two cases of paralytic ileus, which resolved with conservative management. Histopathology confirmed the diagnosis of GIST with low mitotic index of ≤5 mitoses/mm^2^, except for one case where wit was >5 mitoses/mm^2^.

Table 1 summarizes the clinicopathological and features of all six patients.

**Table 1:** Clinicopathological featues of 6 patients with small intestinal GISTs.

Case	Age/ sex	Location	Size	Mitoses per 50 HPF	Clinical presentation	Intraoperative findings	Surgical management	CDC	Adjuvant therapy	IHC	Recurrence^*^
1	55Y/M	Ileum	15x10 cm	≤5	Melena, generalized weakness, dizziness	Large exophytic mass on anti-mesenteric border of mid-ileum, no ascites or peritoneal deposits	Segmental ileal resection with ileo-ileal anastomosis	I	Imatinib 400mg daily	CD117(+), DOG1(+), CD34(+)	None
2	88Y/M	Duodenum	2x4 cm	≤5	Melena, non-bilious vomiting, hypotension	Intraluminal bleeding mass in D3	Open wedge resection of duodenal mass	I	No	CD117(+), DOG1(+), CD34(-)	None
3	75Y/M	Jejunum	2x1 cm	>5	Epigastric pain, constipation, bilious vomiting	Intraluminal mass in distal jejunum, 80 cm from duodenojejunal junction	Segmental jejunal resection with jejuno-jejunal anastomosis	II	Imatinib 400mg daily	CD117(+), DOG1(+), CD34(+)	None
4	70Y/F	Jejunum	5x5 cm	≤5	Malena, tachycardia, hypotension	Exophytic lesion 10 cm from duodenojejunal flexure	Segmental jejunal resection with jejuno-jejunal anastomosis	II	No	CD117(+), DOG1(+), CD34(+)	None
5	55Y/M	Jejunum	15x15 cm	≤5	Acute abdominal pain, abdominal distension, vomiting	Perforated necrotic exophytic mass in proximal jejunum, Moderate ascites	Segmental jejunal resection with jejuno-jejunal anastomosis	II	Imatinib 400mg daily	CD117(+), DOG1(+), CD34(+)	None
6	72Y/M	Duodenum	2x3 cm	≤5	Asymptomatic, incidental diagnosis on imaging	Firm mass on antimesenteric border of D2	Open wedge resection of duodenal mass	I	No	CD117(+), DOG1(+), CD34(+)	None

^*^Recurrence until the study period, followed up 6 monthly by ultrasonography.

## Discussion

GISTs of the small intestine exhibit diverse clinical presentations, largely dictated by tumour size, location, and biological behaviour. In our series, gastrointestinal bleeding was the predominant symptom, mirroring findings from previous reports [11]. In our study, the most frequently observed symptom was the presence of a gastrointestinal bleeding in the form of malena (50%) and abdominal pain (33.3%) followed by a single case of incidental finding (16.6%) similar to a study by Zhou L et al, where gastrointestinal bleeding was the most common symptom (46%) and least number of cases (9.3%) were detected incidentally [12].

Comparing our cases to literature, the sites we encountered were consistent with known distributions of small bowel GISTs, with the jejunum and ileum being the most frequently involved [13]. The mean lesional size was 8.5 cm ranging from 2 to 15 cm, showing a wide range in size at the time of diagnosis, similar to a study by Hamed et al where mean lesional size was 8.76 cm [9]. Two cases that we encountered had a size >10 cm, indicating higher risk of tumour progression, without adjuvant therapy as per a study by Miettinen M et al and modified National Institutes of Health (NIH) criteria [14].

Surgical management remains the mainstay for localized GISTs, and in our series, all patients underwent complete tumor resection. For duodenal GISTs, we performed wedge resections, avoiding more extensive procedures such as pancreaticoduodenectomy, which is sometimes reported in literature for larger or invasive tumours. This in line as per a systemic review and meta-analysis conducted by Shen Z et. Al where they recommend for limited resection to obtain negative margins when the conditions permit, over pancreaticoduodenectomy for duodenal GISTs [15]. For jejunal and ileal tumours, segmental resections with primary anastomosis were performed, following standard surgical principles [16]. Compared to some reports advocating laparoscopic resections, our approach remained open surgery mainly due to emergency presentations and tumour size.

All except one case had a low mitotic rate of ≤5 mitoses per 50 high power field (HPF).

Immunohistochemical staining was performed to characterize the tumour cells. The tumor cells showed positive staining for CD34 in 83% cases while CD117 and DOG1 expression was seen in all the cases.

Adjuvant therapy (Tab Imatinib 400 mg daily) was started in three cases, two cases with size >10 cm and one with HPE revealing mitotic rate of >5 mitoses per 50 HPF. It will be continued for a total period of 5 years as per the PERSIST-5 Clinical Trial [17].

Patients underwent evaluations in the first week post-surgery, followed by assessments at 1, 3, 6, 12, 24, and 36 months using ultrasonography to monitor clinical progress and rule out recurrence. All patients were followed up for a minimum of 12 months. No recurrence was noted in any of the cases during the study period.

Overall, our findings reaffirm that small intestinal GISTs can present with varied and sometimes severe manifestations, necessitating a high index of suspicion for timely diagnosis and intervention. Tumours > 2 cm were resected surgically, open wedge resection for duodenal, and segmental resection for ileal and jejunal GISTs. Adjuvant therapy was started for tumors with high suspicion of recurrence, based on tumor size and mitotic rate.

## Conclusion

Small intestinal GISTs can present with a wide range of symptoms, from minor bleeding to life-threatening complications like obstruction or perforation. The key to managing these tumors effectively is early diagnosis and timely surgical intervention. Our findings highlight the importance of advanced imaging techniques in detecting these tumours and role of surgery as the cornerstone of treatment. For patients with high-risk features, additional adjuvant therapy is beneficial for recurrence.
